# The Prognosis of Pulmonary Tuberculosis
1Read at a meeting of the Society on February 14th, 1906.


**Published:** 1906-03

**Authors:** J. J. S. Lucas


### THE PROGNOSIS OF PULMONARY TUBERCULOSIS.[1]

BY

J. J. S. Lucas, M.D., B.A. Lond.

The subject I have chosen for this short paper is one which has been so much discussed by those more capable of forming opinions than I am, that you may consider it presumption on my part to do more than formulate their opinions, or at most to draw deductions from compilation of statistical results. I however do not intend to do this, but as I have possibly seen more pulmonary tuberculosis than any other serious disease, I am venturing to give you a few of the impressions I have formed and some of the various points I take into consideration in prognosing the future of a phthisical patient. Some of these have undoubtedly been impressed upon my mind by previous reading and confirmed by experience, while others may be erroneous and due to forming conclusions from peculiar groups of cases chance occasionally throws in one's way.

In the first place I will say that I have formed the opinion that it is unwise for a patient who has definitely had pulmonary tuberculosis to ever consider himself a sound man. Once a consumptive, always a consumptive. It is, I think, a very rare thing for a patient who has physical signs of lung tuberculosis and the presence of tubercle bacilli in his sputum to get well and remain perfectly well for many years while attending to his former occupation. A patient under sanatorium treatment may lose all his symptoms and the disease may remain quiescent for a long period, but sooner or later, more especially if he tends to drift into former habits, a relapse occurs which eventually may prove fatal. Within the last few months I have had five patients of this kind. All of them have broken down after having had no active physical signs nor

[1] Read at a meeting of the Society on February 14th, 1906.

symptoms, not even a cough, for more than a year—one for nearly four years. Three of these have got practically well again, one is improving to some extent, while the fifth is rapidly getting worse. I know, of course, that a large percentage of hospital *post-mortems* show healed evidence of tuberculous mischief at an apex, but nearly all of these were not diagnosed, and a large number would have been undiagnosable, due allowance being given for the shrinkage during the contraction in healing. It is certainly rare to find evidence of healed mischief beyond this such as would have been easily diagnosed, as for instance that which would have given infra-clavicular signs.

Having made a diagnosis, one of the most important points in influencing the prognosis is, I think, the appearance of the patient and the configuration of the chest. Anyone by inhaling a sufficiently large dose of bacilli may generate the disease, and I suppose the major portion of sanatorium patients are not of a typical phthisical appearance.

Persons however of the old tubercular and scrofulous types —especially the former, those with a lively temperament and long, narrow chests—unless they are most carefully looked after do somehow or other develop consumption. In these the prognosis is certainly very bad. There seems, as a rule, no way of stopping the disease, and life is prolonged at the most only for a year or so. In these cases the constitution is generally hereditary, and if I examined a patient of this type and found only slight but definite indications of active tubercular mischief I should tell his friends that in spite of treatment the patient would probably gradually lose ground and would never be fit again to resume his previous duties.

On the other hand, in a patient with a well-developed chest and prominent infra-clavicular regions, who had a good family history and who possibly had developed a tubercular patch on one of his lungs after extra hard or unhealthy work, I should be inclined, if there were no signs of great activity, to give the best of prognoses, viz. that with suitable treatment he would lose most, if not all, of his symptoms, and with care would probably live many years, and during that time would be capable of doing a good deal of useful work.

Of early symptoms two of the most suggestive of the further course of the disease are hæmoptysis and dyspepsia. In my experience hemorrhage in the early stages generally indicates a chronic form. Very often, of course, no physical signs indicating phthisis can be found at first, but when they do assert themselves they seem to be of the fibroid type, and the disease tends to run a prolonged course with intervals of good health.

Dyspepsia, on the other hand, is a bad sign. Hygienic treatment may in some cases successfully combat this, but many of the cases cannot be checked, and persistent early indigestion, especially if attended by vomiting, is often the precursor of a progressive fatal illness.

A high temperature at one examination I do not consider of much importance as a rule. The exception is possibly a high temperature—say 104°—in a patient who does not appreciate the fact, and who only feels to be a "bit off colour." This is somewhat akin to the worst forms of typhoid and pneumonia. I have two recent cases in mind which may serve as examples. A doctor's daughter was going away from home to a boarding school, and was dressed ready to go when I chanced to look in. Her mother asked me to see her before she went, as she was not feeling very well. I found her temperature to be 104°, and her sputum on examination showed tubercle bacilli. She only lived six months. Another young fellow came to consult me for general malaise. He had a temperature over 103°, but no physical signs in his lungs. Bacilli were found in his sputum. A trained nurse he was living with at the time pooh-poohed the idea of phthisis, and suggested his doctor was wrong. In spite of treatment, however, he has steadily lost health since, physical signs in both lungs have developed, and he is now in a precarious condition.

The value of a regular temperature chart in treatment is too well known to need comment, and when under treatment an evening temperature two or more degrees above normal persists for a month the outlook is bad. I have certainly seen exceptions to this, but they are rare and may perhaps be said to prove the rule.

From the point of view of prognosis, the pulse to my mind is of more importance than the temperature. The first case of phthisis I saw in private practice was that of a young man with a pulse of 80 and a temperature over 100°. That was five years ago, and he is still alive and at work, and although he occasionally has remissions he is well enough to contemplate marriage (against my advice).

On the other hand, cases I have seen with a pulse more rapid than could be accounted for by the temperature have almost invariably rapidly lost ground.

Of the commoner complications I should like to refer to the influence of laryngeal tuberculosis on the prognosis. From cases I have seen I should divide these cases into at least three varieties: (1) Ulceration of the cords alone, (2) swelling and ulceration of parts above the cord especially affecting the mucous membrane between the arytenoids, the ary-epiglottidean folds, and more rarely the epiglottis, and (3) general ulceration in the late stages of phthisis in which the trachea is often extensively involved with the other portions.

Ulceration of the cord alone does not appear to influence the prognosis. It sometimes occurs at the earliest stages, and I have certainly seen some cases get well.

Perhaps the commonest variety is swelling and ulceration in the outer arytenoid region. This is apt to spread and cause a good deal of huskiness in the voice, a certain amount of pain from involvement of the cartilages, and often dysphagia.

Nothing, as far as I know, can do much good for these, and the interesting point is that the lung physical signs are often masked. In Mr. Lake's book on *Laryngeal Phthisis*, on the front page, is a drawing of a specimen I mounted of extensive laryngeal and tracheal ulceration. I remember perfectly well that a few days before this man died we could only find slight traces of disease in the lungs, but at the *post-mortem* examination both were riddled with cavities. I have a similar case at the present time of a man dying with phthisis with a throat of this kind. Over six months ago his sputum was swarming with bacilli, and since then two doctors have told him his chest was intact. He is developing a few physical signs now, but I feel certain there is much more mischief than these indicate. Why this is so is probably due to small inlet of air into the lungs, and with a throat of this kind I generally conclude that the lungs are much worse than examination of them indicates, and that the prognosis is bad.

Before ending these disjointed remarks I should like to briefly consider how the effect of treatment should influence the prognosis.

If after a few weeks' treatment there seems to be no improvement, but rather deterioration, a change of sanatorium is indicated; and if the result is similar, then after two or three different localities have been tried, it is perhaps better to allow the patient to please himself how he ekes out the last little bit of his wretched existence.

The majority however, I suppose, improve for a time under hygienic treatment anywhere. In some this improvement is only transient, and a maximum is reached in a few weeks, with a steady decline in health afterwards. These patients are very unsatisfactory, and usually in them the prognosis is almost as bad as in those who do not improve at all. Another very annoying group of cases are those who are sent out of sanatoria "*cured*" or "much *improved*," who begin to lose ground directly they come home and resume their necessary duties, although generally under more improved conditions than before. This unfortunately includes a large portion of patients, and I have repeatedly had cases under my charge who have been sent home from sanatoria as well who succumbed in a few months to the disease. The only real test of satisfactory improvement is for patients to be able to live at their homes and to earn a livelihood for months without breaking down.

These, gentlemen, are a few of the points I consider in foretelling the future of a consumptive. They are I know far from complete, and purposely so. I have simply given a few ideas first hand, and have intentionally omitted the influence of concurrent diseases, and the effect on the prognosis of the amount of the tuberculous mischief and its position in the lungs, This is beyond the scope of the paper. The disease is a very deceptive one. Many accidents may happen, and it is unwise to be too definite. To make my remarks quite clear, I will end by enumerating the various points mentioned:—

(1) A phthisical patient should never consider himself sound, at least not for some years, even after loss of all symptoms.

(2) The appearance of the patient, the shape of his chest, and his family history are of the greatest importance in prognosis.

(3) Of early symptoms hæmoptysis by itself is rather a good sign, while dyspepsia is a bad one.

(4) Fever at one examination as a rule does not help the prognosis, but persistent evening temperature for a month is bad.

(5) A rapid pulse is one of the worst of omens.

(6) Of throat complications, ulceration of the cords alone does not indicate much; swelling in the larynx is much more serious, and the physical signs in the lungs are often deceptive in these cases.

(7) The effect of treatment can be gauged only by the condition of the patient several months after he has left the sanatorium, and has undertaken duties he intends to perform for the rest of his life.

